# Evidence-Based Assessment of Genes in Dilated Cardiomyopathy

**DOI:** 10.1161/CIRCULATIONAHA.120.053033

**Published:** 2021-05-05

**Authors:** Elizabeth Jordan, Laiken Peterson, Tomohiko Ai, Babken Asatryan, Lucas Bronicki, Emily Brown, Rudy Celeghin, Matthew Edwards, Judy Fan, Jodie Ingles, Cynthia A. James, Olga Jarinova, Renee Johnson, Daniel P. Judge, Najim Lahrouchi, Ronald H. Lekanne Deprez, R. Thomas Lumbers, Francesco Mazzarotto, Argelia Medeiros Domingo, Rebecca L. Miller, Ana Morales, Brittney Murray, Stacey Peters, Kalliopi Pilichou, Alexandros Protonotarios, Christopher Semsarian, Palak Shah, Petros Syrris, Courtney Thaxton, J. Peter van Tintelen, Roddy Walsh, Jessica Wang, James Ware, Ray E. Hershberger

**Affiliations:** 1 Division of Human Genetics (E.J., L.P., T.A., R.E.H.), Department of Internal Medicine, Wexner Medical Center, The Ohio State University, Columbus.; 2 Division of Cardiovascular Medicine (R.E.H.), Department of Internal Medicine, Wexner Medical Center, The Ohio State University, Columbus.; 3 Department for Cardiology, Inselspital, Bern University Hospital, University of Bern, Switzerland (B.A.).; 4 Department of Genetics, Children’s Hospital of Eastern Ontario, Ottawa, Canada (L.B., O.J.).; 5 Department of Laboratory and Pathology Medicine, University of Ottawa, Ontario, Canada (L.B., O.J.).; 6 Division of Cardiology, Department of Medicine, Johns Hopkins University, Baltimore, MD (E.B., C.A.J., B.M.).; 7 Department of Cardiac-Thoracic-Vascular Sciences and Public Health, University of Padua, Italy (R.C., K.P.).; 8 Clinical Genetics and Genomics Laboratory, Royal Brompton and Harefield NHS Foundation Trust, London, United Kingdom (M.E.).; 9 Department of Medicine, University of California, Los Angeles (J.F., J. Wang).; 10 Cardio Genomics Program at Centenary Institute, University of Sydney, Australia (J.I.).; 11 Victor Chang Cardiac Research Institute, Sydney, Australia (R.J.).; 12 Department of Medicine, University of New South Wales, Sydney, Australia (R.J.).; 13 Division of Cardiology, Department of Medicine, Medical University of South Carolina, Charleston (D.P.J.).; 14 Department of Clinical and Experimental Cardiology, Heart Centre, Amsterdam Cardiovascular Sciences, Amsterdam Universitair Medische Centra, University of Amsterdam, the Netherlands (N.L., R.W.).; 15 Department of Clinical Genetics, Amsterdam University Medical Center location Academic Medical Center, the Netherlands (R.H.L.D.).; 16 Institute of Health Informatics, University College London, London, UK (R.T.L.).; 17 Health Data Research UK London, University College London, UK (R.T.L.).; 18 University College London British Heart Foundation Research Accelerator, London, United Kingdom (R.T.L.).; 19 Cardiovascular Research Center, Royal Brompton and Harefield Hospitals, National Health Service Foundation Trust, London, United Kingdom (F.M., J. Ware).; 20 National Heart and Lung Institute, Imperial College London, United Kingdom (F.M., J. Ware).; 21 Department of Clinical and Experimental Medicine, University of Florence, Italy (F.M.).; 22 Cardiomyopathy Unit, Careggi University Hospital, Florence, Italy (F.M.).; 23 Swiss DNAlysis Cardiogenetics, Dübendorf, Switzerland (A.M.D.).; 24 Cardiovascular Genomics Center, Inova Heart and Vascular Institute, Falls Church, VA (R.L.M., P. Shah).; 25 Invitae Corp, San Francisco, CA (A.M.).; 26 Department of Cardiology and Genomic Medicine, Royal Melbourne Hospital, Australia (S.P.).; 27 Centre for Heart Muscle Disease, Institute of Cardiovascular Science, University College London, London, United Kingdom (A.P., P. Syrris).; 28 Agnes Ginges Centre for Molecular Cardiology at Centenary Institute, University of Sydney, Australia (C.S.).; 29 Department of Genetics, University of North Carolina, Chapel Hill (C.T.).; 30 Department of Genetics, University Medical Center Utrecht, University of Utrecht, The Netherlands (J.P.v.T.).; 31 Medical Research Council London Institute for Medical Sciences, Imperial College London, United Kingdom (J. Ware).

**Keywords:** cardiomyopathy, genetics

## Abstract

Each of the cardiomyopathies, classically categorized as hypertrophic cardiomyopathy, dilated cardiomyopathy (DCM), and arrhythmogenic right ventricular cardiomyopathy, has a signature genetic theme. Hypertrophic cardiomyopathy and arrhythmogenic right ventricular cardiomyopathy are largely understood as genetic diseases of sarcomere or desmosome proteins, respectively. In contrast, >250 genes spanning >10 gene ontologies have been implicated in DCM, representing a complex and diverse genetic architecture. To clarify this, a systematic curation of evidence to establish the relationship of genes with DCM was conducted.

**Methods:**

An international panel with clinical and scientific expertise in DCM genetics evaluated evidence supporting monogenic relationships of genes with idiopathic DCM. The panel used the Clinical Genome Resource semiquantitative gene-disease clinical validity classification framework with modifications for DCM genetics to classify genes into categories on the basis of the strength of currently available evidence. Representation of DCM genes on clinically available genetic testing panels was evaluated.

**Results:**

Fifty-one genes with human genetic evidence were curated. Twelve genes (23%) from 8 gene ontologies were classified as having definitive (*BAG3*, *DES*, *FLNC*, *LMNA*, *MYH7*, *PLN*, *RBM20*, *SCN5A*, *TNNC1*, *TNNT2*, *TTN*) or strong (*DSP*) evidence. Seven genes (14%; *ACTC1*, *ACTN2*, *JPH2*, *NEXN*, *TNNI3*, *TPM1*, *VCL*) including 2 additional ontologies were classified as moderate evidence; these genes are likely to emerge as strong or definitive with additional evidence. Of these 19 genes, 6 were similarly classified for hypertrophic cardiomyopathy and 3 for arrhythmogenic right ventricular cardiomyopathy. Of the remaining 32 genes (63%), 25 (49%) had limited evidence, 4 (8%) were disputed, 2 (4%) had no disease relationship, and 1 (2%) was supported by animal model data only. Of the 16 evaluated clinical genetic testing panels, most definitive genes were included, but panels also included numerous genes with minimal human evidence.

**Conclusions:**

In the curation of 51 genes, 19 had high evidence (12 definitive/strong, 7 moderate). It is notable that these 19 genes explain only a minority of cases, leaving the remainder of DCM genetic architecture incompletely addressed. Clinical genetic testing panels include most high-evidence genes; however, genes lacking robust evidence are also commonly included. We recommend that high-evidence DCM genes be used for clinical practice and that caution be exercised in the interpretation of variants in variable-evidence DCM genes.

## Abstract 2

Supplemental Digital Content is available in the text.

**Clinical Perspective**

**What Is New?**

- Idiopathic dilated cardiomyopathy (DCM), compared with other genetic cardiomyopathies, demonstrates marked locus heterogeneity, with many genes proposed to have a role in the phenotype.
- The complexity of DCM genetic architecture presents challenges to clinical genetic testing and the interpretation of genetic variants in patients and families with DCM.
- The Clinical Genome Resource assembled an international panel of clinicians and scientists with expertise in DCM genetics to conduct a systematic evidence curation to define the relationship of genes with a monogenic role in DCM.

**What Are the Clinical Implications?**

- Although clinical DCM genetic testing panels include an average of ~60 genes, when published evidence for genetic DCM was curated, only 19 genes emerged as high levels of evidence.
- Of 51 genes evaluated, the 19 genes appraised as high-evidence genes are recommended to be routinely used in the genetic evaluation of DCM.
- Rare variants from genes without moderate, strong, or definitive evidence should not be used in clinical practice to predict DCM risk for at-risk family members.

**Editorial, see p 20**

The major cardiomyopathies, diseases of the myocardium, have clinically been classified as hypertrophic cardiomyopathy (HCM), dilated cardiomyopathy (DCM), and arrhythmogenic right ventricular cardiomyopathy (ARVC).^1^ Each has been defined by ventricular structure and function and, in the case of ARVC, supplemented by arrhythmia data. Large families with HCM,^2^ DCM,^3^ and ARVC^4^ provided the basis for discovery of the first genes harboring variants causing these phenotypes.^5–7^ On the basis of further extensive genetic investigations, HCM and ARVC are now well established as predominantly diseases of genes encoding key proteins of the sarcomere^8^ or desmosome,^9^ respectively.

In contrast with the genetic themes observed in HCM and ARVC, DCM has a diverse genetic architecture spanning >10 gene ontologies.^10^ The ultimate explanation for this diversity of genetic architecture in the development of DCM remains incompletely understood, but in large relief, DCM may be considered an end- or final phenotype^11^ that occurs when cellular pathways maintaining force of contraction or ventricular structural integrity become disrupted by pathological variation of genes encoding key proteins.

The number of genes suggested to be relevant for DCM has grown to be very large, in part as a result of this diverse architecture. If we accept the thesis that DCM is an end- or final phenotype and one resulting from myriad possible structural, physiological, or metabolic pathway derangements, DCM candidate genes rightfully number in the hundreds. More broadly, the effort to establish a causal relationship between sequence variants in a gene and a disease is a critical step not only for cardiovascular research but also for the translation of clinical genetics to patient and family-based care.

The National Institute of Health Clinical Genome Resource (ClinGen)^12^ has provided a semiquantitative method to assess the clinical validity of gene-disease relationships.^13^ A panel of cardiologists, genetic counselors, and genetics and laboratory scientists with relevant expertise applied this method to published evidence in DCM, one implemented by other ClinGen cardiovascular domain gene curation panels, including HCM,^14^ ARVC,^15^ thoracic aortic aneurysm,^16^ and the long-QT^17^ and Brugada syndromes.^18^ Here, we report the results of the evidence-based appraisal of genes associated with DCM and the implications of these findings.

## Methods

An international group of individuals from diverse clinical and scientific backgrounds relevant to DCM was assembled as a DCM Gene Curation Expert Panel to implement the ClinGen gene-disease clinical validity classification standards^13^ with specifications to DCM. An initial set of 267 genes was identified from a structured literature search and from gene-disease reference resources (Table I in the Data Supplement). This initial list was triaged to 56 to remove genes that were associated with syndromes or other cardiovascular diseases, had no direct human relevance, or represented candidate genes (Figure I in the Data Supplement). Therefore, genes observed primarily in other phenotypes such as amyloid cardiomyopathy from *TTR* that usually presents with a restrictive cardiomyopathy or mitochondria-related disease were not included because of the strict limitation to curation of only nonsyndromic DCM. The ClinGen precuration process was performed to establish the relevance for DCM, resulting in a final set of 51 genes proposed to have a monogenic role in isolated, idiopathic DCM in humans (Table 1). Additional details on panel membership, operational implementation, and development of the gene list can be found in the Data Supplement. The data that support the findings of this study are published on the ClinGen website (https://clinicalgenome.org/), and gene-specific hyperlinks are provided (Table 1). No formal statistical testing was performed; rather, a systematic analysis of clinical and experimental data was conducted, as described above and in the Data Supplement. No institutional review board approval was required for this work.

**Table 1.**
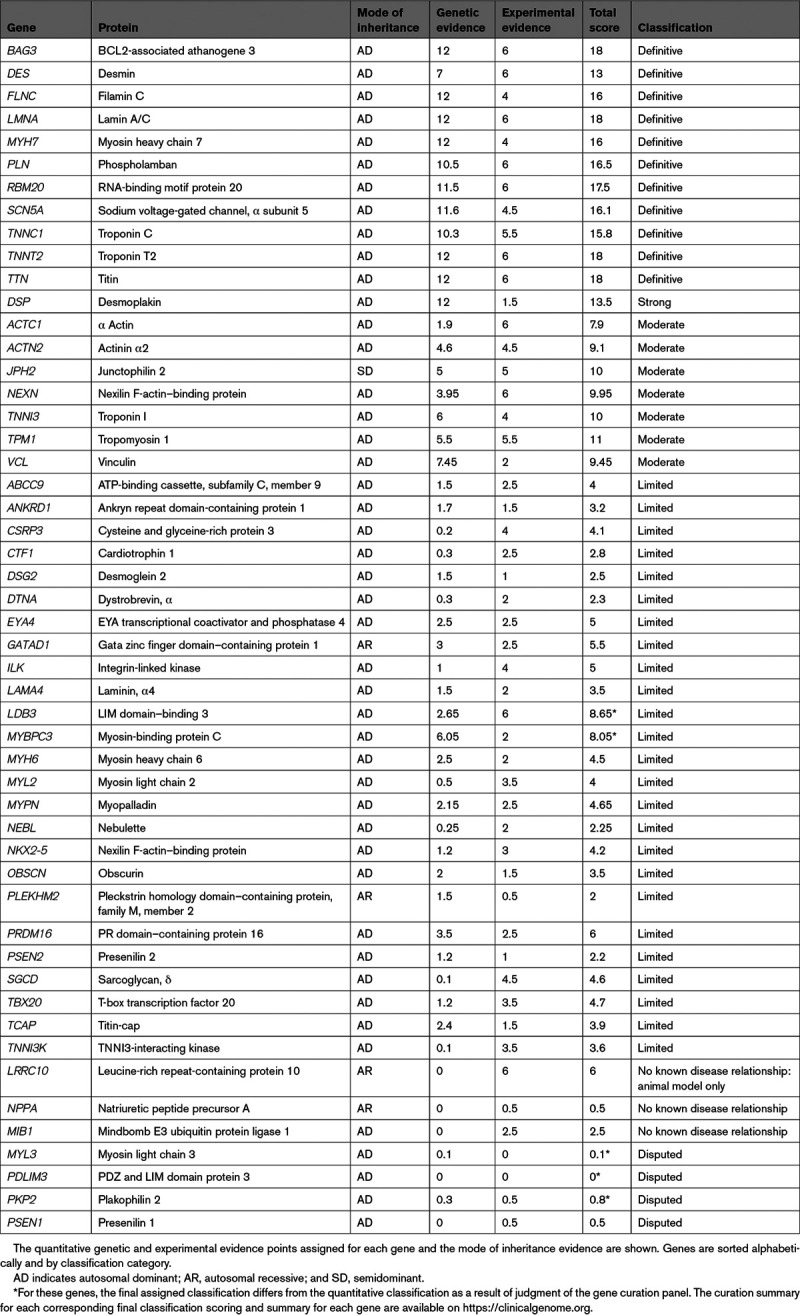
Quantitative Scores and Final Classifications of Genes Curated for Dilated Cardiomyopathy

### Phenotype Definition

The DCM phenotype was defined by systolic dysfunction, conventionally noted as a left ventricular ejection fraction of <50% accompanied by left ventricular enlargement, after other usual clinically detectable causes of cardiomyopathy were excluded. DCM presenting during pregnancy (peripartum or pregnancy-associated DCM) was included because published evidence has demonstrated a genetic background in peripartum or pregnancy-associated DCM that is similar to that in idiopathic DCM.^19,20^ In addition, DCM observed in conjunction with a left ventricular noncompaction phenotype was evaluated and contributed to evidence scores.

Publications used for gene scoring were required to specify how the DCM phenotype was defined and that other usual causes (except genetic) were excluded. In the absence of such specifications, either the data were not scored or the score was reduced from the default points recommended by the ClinGen standard operating procedure (version 7) for the type of variant observed (https://clinicalgenome.org/curation-activities/gene-disease-validity/training-materials/).

### Gene Curation and Evidence Scoring Process

The ClinGen gene curation scoring framework^13^ sums scores for published clinical genetic and experimental laboratory evidence. Members of the panel, trained to curate following the ClinGen protocol, scored published evidence according to the gene-disease clinical validity standard operating procedure version 7. This was presented to the full panel on conference calls to establish an approved clinical validity classification. Genetic evidence was made up of case-level data, including variant evidence and segregation, in addition to case-control data. Variants shown to be absent or to have a minor allele frequency of <0.0001 in gnomAD^21^ (https://gnomad.broadinstitute.org/) were evaluated and scored. Per the standard operating procedure version 7, variant-level evidence was scored on the basis of molecular consequence, missense (up to 0.5 points), and predicted loss-of-function variants (up to 1.5 points), which were adjusted as appropriate if gene-specific DCM-causing mutational mechanisms were established. Individuals or pedigrees with >1 possibly relevant variant in any putative DCM gene were not scored. Experimental evidence was assessed by category (expression data, functional alterations, model systems, and rescue). Additional details are provided in the Data Supplement and were previously published.^13^

The ClinGen clinical validity classifications include strong (12–18 points), moderate (7–11 points), limited (1–6 points), and no known disease relationship (0 points of scorable genetic evidence). The maximum number of genetic evidence points that could be given was 12, and the maximum number of experimental evidence points was 6, for a highest total possible score not exceeding 18 points. “Definitive” was defined as a gene with a strong evidence score with multiple publications over at least 3 years and no contradictory evidence. If the numeric, point-based classification was not considered to reflect the collective assessment of the panel’s clinical and scientific experience, the classification was further discussed, and when applicable, the final classification was modified to a classification reflective of consensus of the panel. Additional details on the clinical validity classification definitions and scoring system are available in the Data Supplement and the standard operating procedure (https://clinicalgenome.org/docs/summary-of-updates-to-the-clingen-gene-clinical-validity-curation-sop-version-7/).

Appraising the strength of the gene-disease relationship precedes the application of clinical variant interpretation standards, as defined by American College of Medical Genetics and Genomics^22^ and modified by ClinGen^23^ and others.^24^ Therefore, the work herein defines the gene set for which current clinical standards of variant interpretation can be applied in practice. With these gene-disease relationships defined, future targeted efforts to refine gene-specific variant curation guidance can take place, with methods defined by the ClinGen variant curation expert panel framework.

### Composition of Clinical Genetic Testing Panels

Sixteen commercially available clinical genetic testing panels curated for DCM were assessed for the presence or absence of the 51 genes curated herein. Panels were identified through a query of the National Center for Biotechnology Information Genetic Testing Registry^25^ by searching the term DCM. The final panel evaluation included targeted DCM multigene panels (Table II in the Data Supplement).

## Results

### Summary of DCM Gene Classifications

**Figure 1.**
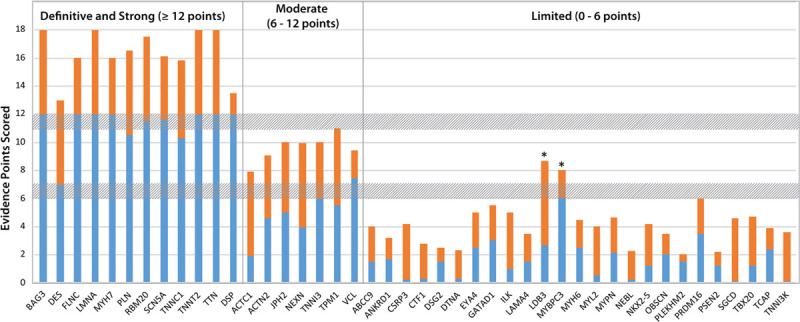
**Quantitative contributions of genetic and experimental evidence to the clinical validity classifications of genes curated for DCM.** The sums of genetic (blue) and experimental (orange) evidence scores are shown for genes classified as having definitive, strong, moderate, or limited evidence of a monogenic relationship with DCM. The 2 genes noted with an asterisk had quantitative scores within the quantitative range for a moderate classification, but a limited classification was assigned at panel review (see text). DCM indicates dilated cardiomyopathy.

**Figure 2.**
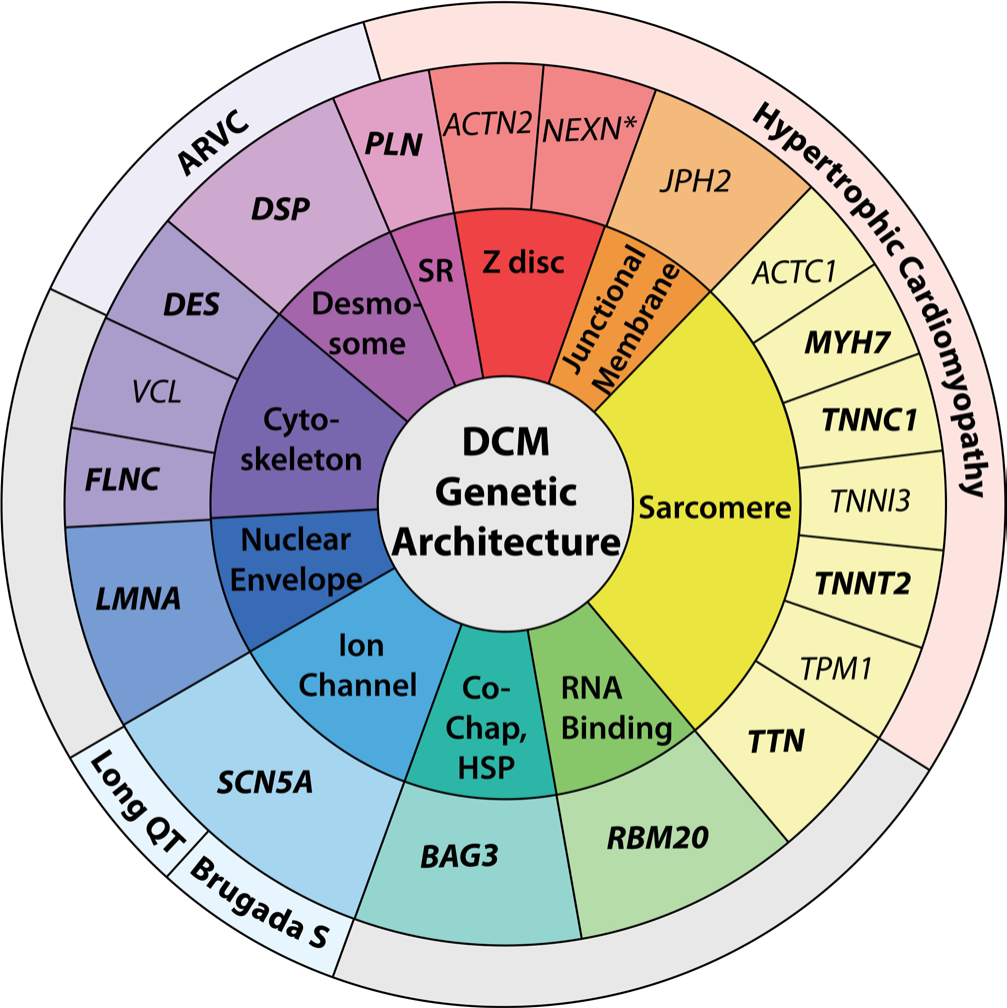
**Genetic architecture of DCM.** The genetic architecture of DCM spans 10 gene ontologies, as shown in the innermost colored text circle. The middle text circle specifies genes classified as strong or definitive (bold text) or moderate (regular text) for DCM, organized by gene ontology. Of the 19 DCM genes shown, 14 have previously been evaluated by other Clinical Genome Resource gene curations for HCM or ARVC and channelopathies, including the long-QT syndrome and Brugada S. Each of these genes has also been classified as moderate, strong, or definitive for these other phenotypes, except for *NEXN*, noted with an asterisk, which has been classified as having limited evidence in HCM. It is expected that with time, as new data emerge that are related to gene-disease relationships in cardiomyopathies and other cardiovascular phenotypes, the structure and orientation of this figure will also evolve. ARVC indicates arrhythmogenic right ventricular cardiomyopathy; Brugada S, Brugada syndrome; Co-Chap, HSP, Co-chaperone, heart shock protein; DCM, dilated cardiomyopathy; HCM, hypertrophic cardiomyopathy; and SR, sarcoplasmic reticulum.

Fifty-one genes were identified as having a role in isolated, idiopathic DCM (Table 1) as described earlier. The appraisal of genetic and experimental evidence resulted in 19 genes with substantial evidence supporting a role in monogenic DCM, including 11 (21%) definitive-, 1 (2%) strong-, and 7 (14%) moderate-evidence classifications (Table 1 and Figure 1) from 10 gene ontologies (Figure 2). It is notable that more than half of genes curated (63%) were determined to be of limited evidence (n=25, 49%), to be disputed (n=4, 8%), to have no known disease relationship (n=2, 4%), or to be supported by animal model data only (n=1, 2%).

### Definitive/Strong Classifications

A total of 12 genes were classified as a definitive or strong relationship (*BAG3*, *DES*, *DSP*, *FLNC*, *LMNA*, *MYH7*, *PLN*, *RBM20*, *SCN5A*, *TNNC1*, *TNNT2*, *TTN*). By definition, strong and definitive classifications represent genes that have a role in DCM that has been clearly demonstrated in the literature over time. Although the ClinGen framework requires a minimum of 2 independent publications to reach a strong or definitive classification, each definitive/strong gene-disease relationship had an abundance of genetic and experimental evidence, ranging from 7 to 12 points and 4.5 to 6 points, respectively, for those classified as definitive. In addition, genes that demonstrated significant enrichment for rare variants in a recently published DCM case-control analysis^26^ emerged as strong or definitive when this evidence assessment was performed.

*DSP* was the only gene with a score ≥12 points that remained classified as strong rather than definitive evidence. Although the criteria for replication over time were met and substantial genetic evidence has been published from rigorously phenotyped cohorts meeting DCM criteria and without clinical evidence of ARVC, curation of experimental evidence presented challenges in scoring due to arrhythmic phenotypes complicating the interpretation of experimental data, resulting in a low experimental evidence score of 1.5 points. The panel ultimately elected to assign a strong classification to *DSP* with opportunities for future curation to reappraise this gene with improved clarification of the interrelationships of *DSP*-related phenotypes, as well as other genes, when the relationship of DCM to arrhythmia adds complexity that exceeds a disciplined approach using current ClinGen curation guidance.

In the curation of *RBM20*, several variants contributing to the genetic evidence score included those within the described hot spot region in exon 9 (amino acids 634 and 636–638).^27^ However, additional missense variants were identified and scored outside of the hot spot region, providing support that variation in addition to that of the exon 9 region may contribute to the DCM phenotype. Scored variation in *TTN* was restricted to premature termination codons, with most located in exons constitutively expressed in the adult heart and in the A band. This was an anticipated finding that provided robust support of pathogenicity because it has been well established that *TTN* alterations of this type are overrepresented in individuals with DCM.^28–32^ Given the complexity of *TTN* variant architecture and the broad contribution of *TTN* variation in DCM beyond the A band represented in our data curation from the published literature, future *TTN* domain- and band-specific curations integrated with expression data should be considered.

### Moderate Classifications

A total of 7 genes were classified as having a moderate level of evidence, namely *ACTC1*, *ACTN2*, *JPH2*, *NEXN*, *TNNI3*, *TPM1*, and *VCL*. The range of genetic evidence points varied widely from 1.9 to 7.45, and experimental evidence varied from 2 to 6 points, with total summative scores ranging from 7.9 to 11 points. Except for 2 genes (*VCL* and *ACTC1*), the contributions of genetic and experimental data to the final classifications were generally balanced (Figure 1). In the case of *ACTC1*, a proportionally higher experimental score (6 points), with fewer substantial published clinical DCM data (1.9 points), contributed to the moderate classification. Conversely, *VCL* has the highest genetic evidence score in the moderate-evidence genes (7.45 points), and although in vitro protein-protein assays, expression studies, and animal models have been published supporting the role of *VCL* in DCM, existing studies could be scored for only 2 points of experimental evidence. With the score of the moderate-classified genes generally totaling at the upper defined range, additional published data and subsequent curations may well result in a future reclassification of these genes to strong or definitive.^33^

### Limited and No Known Disease Relationship Classifications

The majority of genes curated were deemed to have limited or no known disease relationship. The 25 genes with limited evidence (Table 1) had genetic evidence scores ranging from 0.1 to 6.05 points and experimental evidence scores ranging from 0.5 to 6 points. Two genes, *MYBPC3* and *LDB3*, had scores that numerically would have placed them in the moderate-evidence category. However, after review, the panel decided to downgrade the clinical validity classification for both genes to limited. As candidate genes for the DCM phenotype, *LDB3* and *MYBPC3* have been sequenced many times and have accumulated only modest scores, even when considering that they have been targeted in DCM genetic studies for >10 years. Supporting segregation and case-control data are lacking, and the scored genetic evidence was interpreted as circumstantial, additively placing them in a higher category over time regardless of the absence of strongly supportive data. Therefore, the quantitative classification exceeded the panel’s overall assessment of the clinical relevance of these genes in idiopathic DCM. Without these genes, the range of limited evidence scores is 0.1 to 3.5 and 0.5 to 4.5 for genetic and experimental evidence, respectively.

*EYA4* was curated for DCM and hearing loss as the disease entity (MONDO:0011541). Although the filtering of the initial gene list excluded genes related primarily to a syndrome affecting systems beyond the cardiovascular system, the hearing loss phenotype observed in *EYA4* can be quite subtle and may not be clinically apparent at the time of the genetic evaluation of DCM. Therefore, the panel curated this gene separately for its role in the DCM phenotype, with or without hearing loss, and it was classified as having limited evidence.

Genes determined to have no known disease relationship, formerly referred to as no reported evidence, include *LRRC10*, *NPPA*, and *MIB1*. This classification indicates that the gene does not have human genetic evidence suggesting a causal role in monogenic DCM. Although many candidate genes with no currently known relationship with human DCM were removed during the development of the initial gene list, these genes emerged as a result of the degree of experimental data suggesting a role in DCM development. Although these genes have experimental evidence supporting a relationship with the DCM phenotype, there was an absence of human genetic evidence meeting DCM criteria defined for this curation effort (*MIB1*, *NPPA*). Of note, in the curation of *LRRC10*, published experimental data^34^ included nonhuman model organisms, and it accumulated an experimental evidence score much higher (6 points) than *NPPA* (0.5 points) and *MIB1* (2.5 points). In addition, although human genetic evidence for *LRRC10* had been published, the data were excluded after panel review. Nonetheless, because of the compelling *LRRC10* animal model evidence, the “animal model only” tag was added to the no known disease relationship classification. If human data emerge for *LRRC10*, future curation may result in reclassification.

### Disputed Classifications

The disputed classification was assigned when available evidence was insufficient and a question was raised about relevance to a monogenic causation of DCM. Four genes (*MYL3*, *PDLIM3*, *PKP2*, and *PSEN1*) were disputed after curation and panel discussion. Each of these genes had minimal genetic evidence that was able to be scored after review, mainly because the frequency of the few variants reported in the general population exceeded the defined minor allele frequency cut point of 0.0001. Furthermore, in a recently published case-control analysis of DCM genes, *PKP2* and *PDLIM3* variants were not enriched in cases compared with a control population.^26^ Whereas *PKP2* has previously been curated as a definitive-evidence gene for the ARVC phenotype with several rare, predicted loss-of-function variants published in ARVC cases,^35^ when the clinical data for a strict DCM phenotype were curated, the currently available literature provided only a few missense variants and was considered insufficient. Ultimately, the panel concluded that the current evidence for the *MYL3*, *PDLIM3*, *PKP2*, and *PSEN1* genes was not sufficient to support a causative, monogenic relationship with the DCM phenotype.

### Composition of DCM Genes on Clinical Genetic Testing Panels

**Figure 3.**
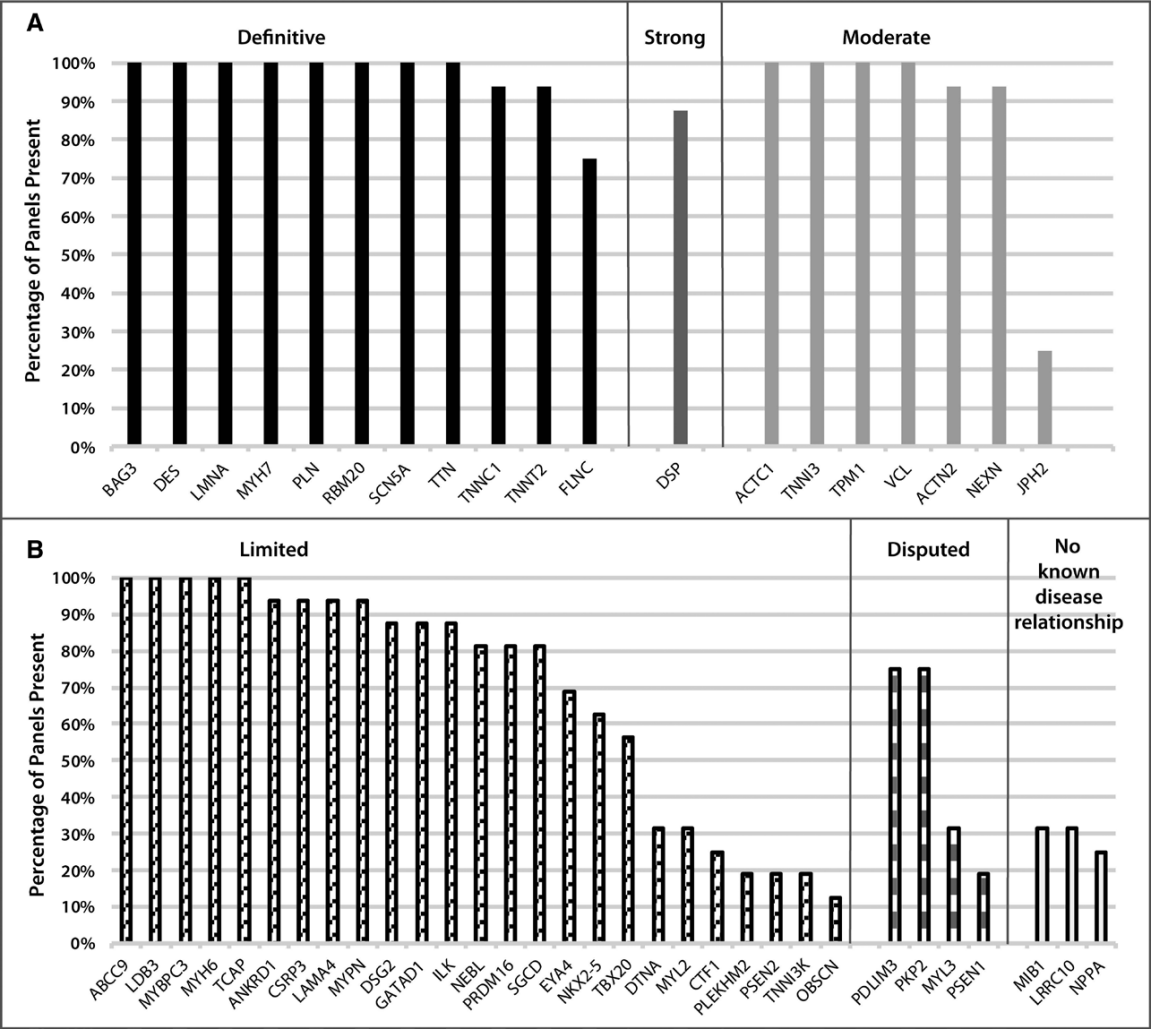
**Curated genes on clinically available DCM genetic testing panels.** The percentages of DCM genetic testing panels that include the genes curated for DCM herein are shown for 16 commercial laboratories identified on the National Center for Biotechnology Information genetic testing registry. Genes are grouped by clinical validity classification, ranging from definitive, strong and moderate (A) to limited, disputed, and no known disease relationship (B). DCM indicates dilated cardiomyopathy.

Sixteen commercially available clinical genetic testing panels were evaluated for DCM gene inclusion (Figure 3). On average, evaluated panels contained a total of 64 genes, with the total number of genes ranging from a minimum of 37 to a maximum of 123 genes. A total of 229 unique genes were represented among the panels evaluated, 94 of which were not included on the original list herein because they have not been asserted as primary DCM genes in the literature or public databases but rather are part of other disease spectra (skeletal myopathy, metabolic/mitochondrial disease, etc, that were excluded, as noted in the Methods). Of all panels, 50% (n=8) offered testing for ≥75% of the genes curated for DCM. Eight of the 11 definitive genes appeared on all panels, with *TNNC1* and *TNNT2* present on 95% and *FLNC* present on 75% of panels. The observation that *FLNC* is included on only 75% of clinical testing panels may be explained by its relatively recent emergence in DCM, with a first major publication in 2017.^36^ Except for the moderate-classified *JPH2*, which appeared in only 25% of panels, genes classified as definitive, strong, or moderate appeared on a majority of evaluated panels (75%–100%). The presence of limited genes ranged widely (13%–100%), with *ABCC9*, *LDB3*, *MYBPC3*, *MYH6*, and *TCAP* present on all evaluated panels and *CTF1*, *PLEKHM2*, *PSEN2*, *TNNI3K*, and *OBSCN* present on the fewest (12%–25%). In addition, some disputed genes were represented more commonly than those with limited evidence, for example, *PDLIM3* and *PKP2*, both of which were present on 75% of evaluated panels.

## Discussion

This study conducted a systematic review and curation of published evidence for genes considered relevant for monogenic DCM. Winnowed to 51 genes for curation from an initial list of 267 candidates, each gene was scored according to the established ClinGen framework^13^ used previously for other cardiovascular genetic conditions.^14–18^ Twelve genes were found to have definitive or strong relationships, and 7 had moderate evidence for a monogenic cause of DCM. These 12 high-evidence genes are consistent with the findings of previous case-control studies,^26,32^ with the genes with variants enriched in DCM cases also found to be of high evidence when the ClinGen curation framework was applied. Of the remaining 32 genes, 25 were determined to be limited, and 7 were disputed or assigned as having no known disease relationship because of a lack of human evidence. To the best of our knowledge, this effort represents the first standardized curation of evidence implicated in the monogenic cause of DCM and provides guidance to clinicians for testing strategies in the genetic evaluation of DCM.

This work underscores the diverse genetic architecture of DCM and illuminates the intersections of genes relevant for DCM with other well-established cardiovascular gene-phenotype relationships. It also illustrates the complexity of DCM genetics. Several genes curated as definitive for other cardiomyopathy or arrhythmia phenotypes were also scored as definitive for DCM (Figure 2). One of the most prominent arrhythmia genes, *SCN5A*, definitive for DCM, has also been classified as a definitive gene in long-QT type 3^17^ and Brugada^18^ syndromes. Although the precise molecular mechanisms that result in a DCM versus an electrophysiological phenotype attributable to an *SCN5A* variation remain incompletely understood, the *SCN5A* clinical and experimental evidence for DCM achieved a definitive classification. Genes encoding proteins of the desmosome have also been proposed to be relevant for DCM,^37^ and *DSP*, *DSG2*, and *PKP2*, considered definitive when curating an ARVC phenotype strictly defined by the Task Force criteria,^38^ had various degrees of evidence when curated strictly for DCM.^15^ In the case of DCM, *DSP* was scored as strong and may likely move to a definitive classification in future re-evaluation. *DSG2* was scored as limited, and the lack of monogenic DCM evidence for *PKP2* resulted in a disputed classification. Additional human clinical genetics data from well-phenotyped cohorts will be needed to further clarify the relevance of these genes for DCM. Two sarcomere genes, *MYH7* and *TNNT2*, established as definitive for HCM, were also definitive for DCM. Three other definitive genes for HCM, *TNNI3*, *TPM1*, *ACTC1*, were considered of moderate evidence for DCM but may emerge as strong or definitive genes for DCM with additional evidence. Conversely, *TNNC1*, definitive for DCM, was scored as moderate evidence for HCM.^14^

Other genes scored as definitive with evidence principally from the DCM phenotype further highlight the diverse genetic architecture of DCM, in contrast to ARVC and HCM (Figure 2). Most notable is *TTN*, an enormous scaffolding protein of the sarcomere, which contributes the most cases of DCM.^28,29^ For HCM and ARVC, *TTN* was classified as limited.^14^ *LMNA*, encoding a protein of the inner nuclear membrane that exhibits striking pleiotropic effects in skeletal muscle, adipose, and other tissues, was considered definitive for DCM but limited for ARVC. *RBM20*, which encodes an RNA-binding protein that regulates RNA splicing, was scored as definitive for DCM and has no other phenotypic representation beyond DCM. *FLNC*, an actin cross-linking protein widely expressed in cardiac and skeletal muscle, was also classified as definitive in DCM. Additional genes with moderate evidence in association with DCM also illustrate the diversity of DCM genetic architecture (Figure 2). Alternatively, genes with insufficient evidence in DCM have been appraised as high evidence in HCM or ARVC (Table 2).

**Table 2.**
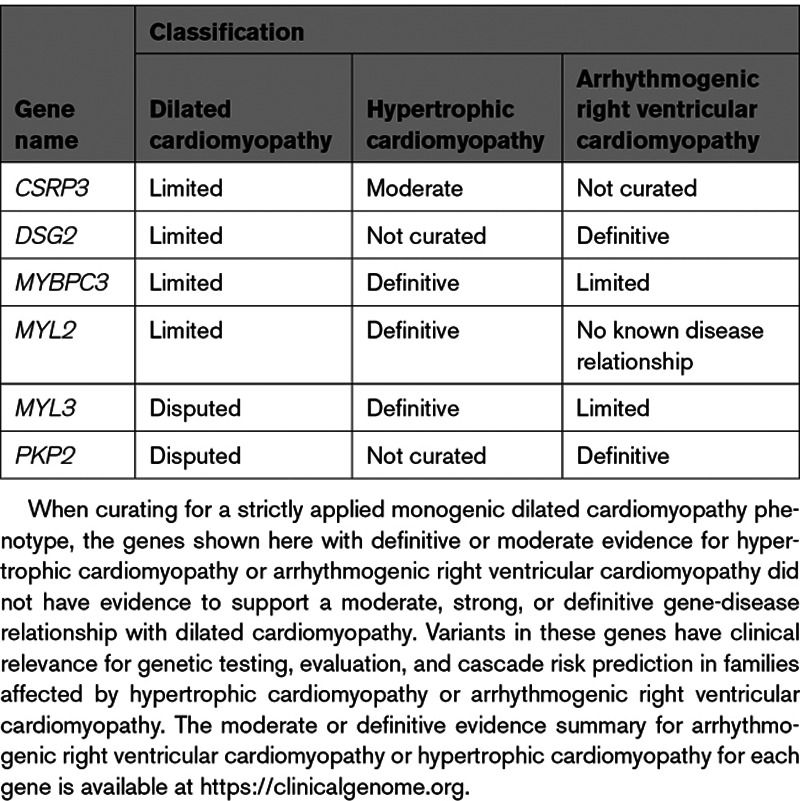
Genes Classified as Limited or Disputed for Dilated Cardiomyopathy With Definitive or Moderate Classifications for Hypertrophic Cardiomyopathy or Arrhythmogenic Right Ventricular Cardiomyopathy

The work presented is an important step forward in describing the genetic architecture of monogenic, primarily adult-onset, nonsyndromic DCM. However, despite this progress, a great deal of work remains to more fully understand the genetic basis of DCM. Even after a rigorous evaluation of variants identified in DCM genes, a pathogenic or likely pathogenic classification can be established in only a minority of patients with DCM, estimated at 20% to 35%.^39^ This modest genetic testing sensitivity is observed even in the case of multigenerational families who have multiple affected members, which on its face supports an underlying genetic cause. The reason for this low testing sensitivity even for familial DCM remains unclear. This appears to be related in part to the already established locus heterogeneity, with a substantial number of genes already established as relevant. It is notable that almost all genes account for only a small percentage of cause, exceptions including *TTN* explaining up to 15% to 20%,^29^ *LMNA* in up to 4% to 6%,^40^ and *MYH7* up to ≈3%^26^ of DCM cases. Whether any of the 25 genes attributed to have limited evidence will emerge as moderate, strong, or definitive remains to be determined. It is also possible, if not likely, that additional genes considered novel to DCM, even from yet-to-be-included ontologies, remain to be identified.

Alternatively, genetic mechanisms exceeding those considered monogenic could well be at play. Previous evidence has suggested that some proportion of DCM may have an oligogenic or polygenic basis,^24,40–42^ but a DCM phenotype confounded by >1 rare variant was not accounted for under the ClinGen framework^13^ and therefore not scored because all curations assumed a classic (monogenic) mendelian paradigm. According to preliminary data, this may be an important focus for ongoing effort. Other types of genetic variation that to date have received less investigation in DCM, including promoter variants, common variants, or structural variants exceeding in size those able to be detected by next generation sequencing, also may be relevant to define DCM genetic cause. The recent use of larger DCM cohorts has revealed common variants that modulate the DCM rare variant phenotype studied here,^43^ which underscores the utility and need of assembling even larger cohorts of patients and families with DCM for study. Furthermore, a majority of contemporary gene-disease association studies represent populations of primarily European ancestry. There is a marked deficit in the understanding of the DCM genetic architecture in non-European cohorts. The DCM Precision Medicine Study^24,44^ is an ongoing effort specifically designed to address this knowledge gap. Future research efforts should continue to focus on populations of diverse racial and ethnic backgrounds in order to understand DCM genetic architecture more fully.

The findings of this curation effort are also relevant for family-based clinical genetics care of patients with DCM.^45^ Establishing genetic risk of DCM in family members as a component of a genetic evaluation presents considerable opportunity for disease mitigation and prevention. Genetic testing is a central component of a DCM genetic evaluation, and most commercially available DCM gene panels test several dozen genes, well exceeding the 19 genes curated here as definitive, strong, or moderate. In the setting of a DCM phenotype, variants reported in genes beyond these 19 can at most be classified only as variants of uncertain significance, and in the cases of disputed genes or those with no known disease relationship, identified variants are not able to reach a clinical classification. This is appropriate and nearly unavoidable because these genes have clinical validity yet to be defined for DCM. According to the evidence presented, moderate-evidence DCM genes may eventually gain sufficient clinical and experimental support to be assigned as strong or definitive for DCM at the time of future curation. Whether the many limited-evidence genes will also accumulate sufficient clinical or experimental data over time in order to emerge as strong or definitive remains to be determined.

Nevertheless, some genes strictly curated for DCM as limited or even disputed have been classified as definitive or moderate evidence for HCM or ARVC (Table 2). We also note that evaluating variants in these genes to assess gene-disease relationships, in individuals carefully selected from published accounts following strictly applied clinical criteria for DCM, HCM, or ARVC by this and other ClinGen gene curation groups, at times does not represent the clinical reality of patients who cannot be so easily categorized phenotypically. Such overlap cases with regard to DCM (eg, DCM and HCM, or DCM and ARVC) represent vexing real-life cases for cardiovascular clinicians and genetics professionals alike, for example, where biventricular ARVC can be phenotypically indistinguishable from DCM, and the initial phenotype assignment may be based on a nuanced interpretation of the available clinical evidence, as well as the specialty training and previous experience of the cardiovascular clinician. Moreover, cardiovascular clinicians and genetics professionals who conduct and interpret genetic testing need to understand that such genes (Table 2) represent evolving clinical cardiovascular genetic practice and that a pathogenic or likely pathogenic variant curated for 1 phenotype with a moderate, strong, or definitive gene-disease relationship classification, but considered low evidence or irrelevant for another phenotype, at times should trigger a re-evaluation. Variant classification is dynamic and probabilistic, with reconsideration of an initial classification occurring in light of additional proband- or family-based clinical data. This iterative process is a common practice to cardiovascular clinical and genetics professionals who routinely encounter such challenges.

As noted, one of the most significant issues for the majority of families is that with current genetic testing the genetic cause of DCM often remains elusive.^39^ In addition to marked locus heterogeneity, the modest testing sensitivity may be explained in part by the marked allelic heterogeneity that also confounds variant interpretation, because many DCM variants are private or, even if previously observed, lack sufficient data to support pathogenicity using the current stringent standards for variant adjudication.^22–24^ Moreover, as previously mentioned, nonmendelian mechanisms, even if clearly defined, may not be easily integrated into conventional approaches to variant interpretation.^22^

As acknowledged by other ClinGen cardiovascular domain gene curation panels,^14–18^ clinical genetic testing panels feature many genes classified as limited, disputed, or with no known disease relationship with the phenotype of interest. Current variant adjudication guidance^22–24^ is intended for the clinical interpretation for the monogenic cause of disease in order to translate genetic test results into information to be applied to clinical, family-based care.^45^ Although many genes have been suggested to have a relationship with DCM as shown in the initial expansive list, only a minority were identified to have a possible monogenic role in idiopathic DCM. The rationale for commercial sequencing panels to include genes for DCM even beyond the 44 limited-, moderate-, strong-, and definitive-evidence genes curated here is unclear, although some represent syndromic conditions in which the DCM phenotype may be observed as a feature of the syndrome. It is possible that knowledge gained about candidate genes might benefit the research community for the purpose of discovery, or a more expansive gene list may represent other interests of commercial laboratories. Nevertheless, the inclusion of genes lacking even moderate evidence of a gene-disease relationship contributes to uncertainty in clinical care for patients and providers. This also creates the potential for misapplication of genetic information in the care for patients with DCM and their at-risk family members.^45^

### Implications for Clinical Care

The results of this analysis indicate that pathogenic and likely pathogenic variants in the 19 higher-evidence genes (definitive, strong, moderate) can reasonably be used for diagnostic and predictive purposes in the management of patients and families with monogenic DCM. According to this contemporary curation of available evidence, these 19 genes should be included in DCM clinical genetic testing panels and used to predict DCM risk in asymptomatic individuals. However, it is unclear whether genes assigned to limited, disputed, or no known disease relationship classifications will emerge as mendelian causes of DCM as more evidence is published. Although acknowledging that genes with insufficient evidence may be clinically relevant for other cardiomyopathies (Table 2), analysis of the currently available evidence suggests that variation in these genes in DCM is largely uninformative in isolation. With the possible exception of a large family with ample opportunity for segregation analysis, variants in genes not classified as moderate, strong, or definitive evidence will seldom be interpretable for DCM and therefore are not recommended to be used for cascade evaluation for DCM risk. Inclusion of such genes of uncertain significance on clinical testing panels for a strict DCM phenotype should be done with caution and ideally in the context of an expert multidisciplinary team to avoid misapplication.

### Conclusions

In this study, an evidence-based curation of published literature evaluating the clinical validity of the monogenic relationship with DCM was performed. Of 51 genes, 12 were classified as definitive or strong evidence and 7 as moderate evidence. These 19 genes provide a solid foundation for clinical care. The remaining genes, classified as limited evidence or no known disease relationship, have limited clinical utility but may provide valuable information for investigators as additional evidence in support of genetic cause of DCM is sought. Several of the genes classified as definitive for DCM also have been classified as definitive for other distinct cardiomyopathy or arrhythmia phenotypes, underscoring the unique and diverse genetic architecture of DCM. Despite this, the current sensitivity of genetic testing in DCM of only 20% to 35% emphasizes the need for continued efforts to more fully understand the genetic basis of DCM, whether from known candidate genes or those not yet understood to be relevant or from genetic mechanisms yet to be more fully described.

## Acknowledgments

The authors thank Stephanie Schulte, MLIS, Associate Professor and Head of Research and Education Services at the Health Sciences Library at The Ohio State University, for her assistance in developing a systematic and comprehensive approach to developing the initial gene list from the OMIM, Gene, and GenBank databases.

## Sources of Funding

This publication was supported by the National Human Genome Research Institute of the National Institutes of Health (NIH) under award U41HG009650 and by a parent award from the National Heart, Lung, and Blood Institute of the NIH under award R01HL128857 (Dr Hershberger), which included a supplement from the National Human Genome Research Institute. The content is solely the responsibility of the authors and does not necessarily represent the official views of the NIH. Dr Semsarian is the recipient of a National Health and Medical Research Council Practitioner Fellowship (No. 1154992). Dr Lumbers is supported by a UK Research and Innovation Rutherford Fellowship hosted by Health Data Research UK (MR/S003754/1) and by the BigData@Heart Consortium funded by the Innovative Medicines Initiative-2 Joint Undertaking under grant agreement 116074. P. Shah is supported by a National Heart, Lung and Blood Institute career development award (1K23HL143179). Dr van Tintelen received funding from Netherlands Cardiovascular Research Initiative, an initiative supported by the Dutch Heart Foundation (CVON projects 2015-12 eDETECT, 2018-30 PREDICT2). J.S. Ware is supported by the Wellcome Trust [107469/Z/15/Z], Medical Research Council (UK), National Institute for Health Research Royal Brompton Cardiovascular Biomedical Research Unit, and the National Institute for Health Research Imperial College Biomedical Research Center. Dr Ingles is the recipient of an National Health and Medical Research Council Career Development Fellowship (No.1162929).

## Disclosures

The following authors have contributed to the literature and/or actively participate in research related to gene curation, gene discovery, and genetic testing: Drs Lumbers, Mazzarotto, Judge, Walsh, Lekanna Deprez, Ai, Ingles, Semsarian, A. Morales, E. Jordan, L. Peterson, Cynthia James, Palak Shah, Petros Syrris, Jessica Wang, and James Ware. The following authors are an employee, trainee, or consultant for a commercial laboratory offering genetic testing, genetic counseling, and/or therapeutics related to dilated cardiomyopathy: E. Brown, Dr Judge, A. Morales, B. Murray, and J. Fan. J. Ware and Dr Ingles are consultants for Myocardia, Inc. The other authors report no conflicts.

## Supplemental Materials

Expanded Methods

Data Supplement Figure I

Data Supplement Tables I and II

## Supplementary Material


