# Supplementary material for: Evidence-Based Assessment of Genes in Dilated Cardiomyopathy
Source: Circulation. 2021 May 5;144(1):7–19. doi: 10.1161/CIRCULATIONAHA.120.053033 (PMC8247549; doi:10.1161/CIRCULATIONAHA.120.053033)
Supplement: Supplementary file 1 [file cir-144-07-s001.pdf]

SUPPLEMENTAL MATERIAL

Supplemental Methods

Panel membership and workflow

DCM gene curation activities were carried out by an international, multi-site Panel of individuals with expertise in laboratory science, molecular diagnostics, research, and/or clinical care in idiopathic DCM. The Panel, termed "The DCM Gene Curation Expert Panel," a sub-working group of the ClinGen Cardiovascular Clinical Domain Working Group, was composed of 34 individuals from over 20 institutions representing 7 countries (United States, Canada, Netherlands, Australia, United Kingdom, Switzerland, and Italy). Using the ClinGen standard operating procedure (SOP) version 7 (https://clinicalgenome.org/docs/summary-of-updates-to-the-clingen-gene-clinical-validity-curation-sop-version-7/), curators scored available evidence and prepared a presentation for full Panel review, discussion, and approval during a biweekly conference call between September 2019 and October 2020 to establish a final classification. Curated data was entered into a web interface, the Gene Curation Interface (GCI), to organize and record evaluated evidence.

Development of the DCM gene list

To develop a comprehensive list of genes thought to have a role in the development of DCM in humans, a comprehensive search was conducted in three National Center for Biotechnology Information database resources in October 2017: Gene (https://www.ncbi.nlm.nih.gov/gene), Online Mendelian Inheritance in Man (OMIM)[1] (https://www.ncbi.nlm.nih.gov/omim), and GenBank[2] (https://www.ncbi.nlm.nih.gov/genbank/). Starting with Gene, the search terms "dilated cardiomyopathy" OR "cardiomyopathy, dilated" were applied and limited to Homo Sapiens to identify 199 genes. A subsequent search in OMIM

and GenBank using the same search terms, limiting to Animals and Genomic DNA/RNA as the molecule type, identified a list of 204 genes. This initial list was further expanded when adding additional genes not identified in the database query from an expansive DCM gene list that had been previously published[3], which added 63 genes. The full initial list included 267 genes.

Each gene was then manually evaluated by performing a PubMed search of the "[GENE]" AND "dilated cardiomyopathy" in addition to "[GENE]" AND "cardiomyopathy, dilated" to identify relevant publications proposing a role in the development of idiopathic DCM in humans (May 2018). Genes were removed for the following reasons: those specified as possible candidate genes without supporting animal or human genetic evidence; those that did not have published evidence in human idiopathic DCM; those described as primarily part of a syndrome; and those primarily observed in other non-DCM cardiovascular diseases. Following the application of these criteria, 56 genes remained.

Genes implicated in more than one disease underwent precuration to confirm the DCM disease entity and mode of inheritance for each gene curated (https://clinicalgenome.org/working-groups/lumping-and-splitting/). Precurations were performed to identify the disease entity and mode of inheritance. Precurations were shared with the Panel for review prior to formal preparation and scoring of evidence to assign a gene-disease validity classification. During this process, five genes were removed from the curation schedule due to syndromic disease entity (*SDHA*), lack of sufficient evidence specific to the DCM phenotype as defined for this curation effort (*DSC2*, *GATA4*, *GATA6*), or a different cardiomyopathy sub-type as the primary disease entity without a clear assertion for an isolated DCM phenotype in a single gene-disease relationship (*ALPK3*). The remaining 51 genes represent the final gene list (June 2019) (Figure S1).

Five of the genes on the final list were identified as predicted to be classified as definitive (*BAG3*, *LMNA*, *MYH7*, *TNNT2*, and *TTN*) and therefore underwent an expedited curation, requiring the review and approval of two Panel members of the evidence review in an offline mechanism, followed by review and approval of the evidence summary by the full Panel prior to publishing. In addition, two genes had been recently curated by the Hypertrophic Cardiomyopathy Gene Curation Expert Panel (HCM GCEP) for an applicable disease entity, intrinsic cardiomyopathy (MONDO:0000591), and mode of inheritance prior to the DCM gene curation project (*PLN*, https://search.clinicalgenome.org/kb/gene-validity/8772; *ACTN2* https://search.clinicalgenome.org/kb/gene-validity/8772)[4]. The other 44 genes underwent the standard curation process.

Curation of Published Evidence

Evaluation of genetic evidence included publications presenting clinical data for patients, families, and large cohorts that could be consider for scoring of individual variants, segregation in pedigrees, and case-control analyses. A minor allele frequency (MAF) cut-off for the maximum credible frequency to have a pathogenic effect was calculated using an allele frequency calculator which integrates disease prevalence, mode of inheritance, genetic and allelic heterogeneity, and penetrance (https://www.cardiodb.org/allelefrequencyapp/).[5] We sought to produce a conservative estimate consistent with Mendelian, autosomal dominant disease, occurring at a prevalence 1/250 individuals in the population[6]. While reduced penetrance is commonly observed in DCM, penetrance estimates are not widely available. Further, the penetrance of the genes included in the curation list are anticipated to vary widely, therefore, estimates of 20-40% penetrance were considered. DCM is also highly heterogeneous, with no single variant causing more than 1-2% of DCM cases. Taking these values into consideration, a

maximum credible population allele frequency of 0.0001 was generated, consistent with the clinical standard[7] for monogenic DCM. Variants with a MAF <0.0001 were scored in the curation of genetic evidence.

Experimental evidence was assessed by category (expression data, functional alterations, model systems, and rescue). Expression evidence prioritized studies of human cardiomyocytes or animal models as an alternative when human models were not available. Model systems assessing the disruption to the gene in consideration demonstrated a phenotype suggestive of DCM, and rescue models using cell-culture or non-human animal models with DCM could be rescued by restoring wild-type gene product. Experimental data only evaluating rare variants with some evidence of impact were scored.

Genes with a score reaching a strong classification that demonstrated replication over time in the literature, as defined by at least three years since original publication and at least two independent supporting publications, were classified as definitive. Limited evidence genes lack substantial evidence supporting the gene-disease relationship, however, data challenging the relationship is also not present, and therefore while the role in monogenic disease is not supported based on currently available evidence, the clinical relevance of these genes is not ruled out. Genes were classified as disputed when evidence was deemed insufficient and panel opinion and/or literature questioned the biological relevance of the gene in the disease. Genes classified as no known disease relationship and animal model only represent candidate genes that do not yet have sufficient human data to evaluate for a clinical classification.

Supplemental Figure I

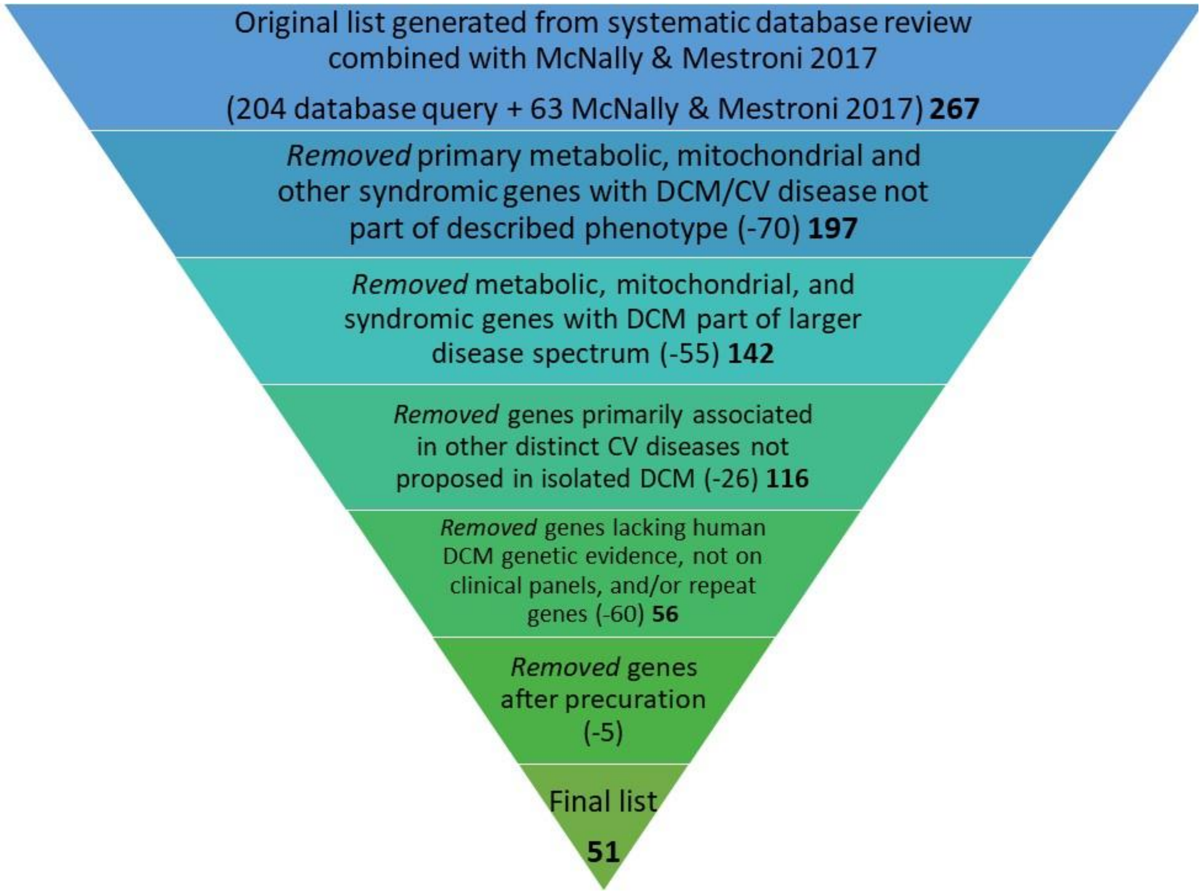

Figure SI. **DCM gene list filtering.** An original list of 267 genes, generated by OMIM, Gene, and GenBank database queries combined with a previously published DCM gene list[3], was filtered down to a list of 51 genes indicated in humans with isolated, idiopathic DCM that proceeded through the gene curation process. CV = cardiovascular; DCM = dilated cardiomyopathy.

ORIGINAL LIST (OMIM; GENE; GenBank)	McNally 2017 (PMID 28912180)	REMOVED GENES (ALL)	REMOVED- MT/METABOLIC/ SYNDROMIC	REMOVED- NOT IDIOPATHIC DCM (OTHER CM/CV DZ)	REMOVED - NOT IDIOPATHIC DCM (NON-CV DZ/syndrome)	REMOVED - PRECURATION	REMOVED - OTHER	FINAL LIST
**ABCC9**	**ABCC9**							**ABCC9**
ACADVL		ACADVL	ACADVL					
ACE		ACE			ACE			
**ACTC1**	**ACTC1**							**ACTC1**
**ACTN2**	**ACTN2**							**ACTN2**
ADIPOQ		ADIPOQ	ADIPOQ					
ADORA1		ADORA1			ADORA1			
ADRA2C		ADRA2C		ADRA2C				
ADRB1		ADRB1		ADRB1				
ADRB2		ADRB2			ADRB2			
	AGL	AGL	AGL					
AGT		AGT			AGT			
AGTR1		AGTR1			AGTR1			
ALMS1	ALMS1	ALMS1	ALMS1					
	ALPK3	ALPK3				ALPK3		
**ANKRD1**	**ANKRD1**							**ANKRD1**
ANO5		ANO5	ANO5					
AR		AR			AR			
**BAG3**	**BAG3**							**BAG3**
BCL2		BCL2					BCL2	
BMP4		BMP4			BMP4			
	BRAF	BRAF	BRAF					
BTNL2		BTNL2		BTLN2				
	CACNA1C	CACNA1C		CACNA1C				
CACNG8		CACNG8					CACNG8	
	CALR3	CALR3					CALR3	
CASK		CASK			CASK			
	CASQ2	CASQ2		CASQ2				
CASZ1		CASZ1					CASZ1	
	CAV3	CAV3		CAV3				
CAVIN4	(MURC)	CAVIN4/MURC					REPEAT (MURC)	
CD34		CD34					CD34	
CD40LG		CD40LG			CD40LG			
CD46		CD46			CD46			
CDH2		CDH2	CDH2					
CHGA		CHGA					CHGA	
	CHRM2	CHRM2					CHRM2	
CHRM3		CHRM3			CHRM3			
CMD1B		CMD1B					CMD1B	
CMD1H		CMD1H					CMD1H	
CMD1K		CMD1K					CMD1K	
CMD1Q		CMD1Q					CMD1Q	
	CPT2	CPT2	CPT2					
CRP		CRP					CRP	
CRYAB	CRYAB	CRYAB	CRYAB					
**CSRP3**	**CSRP3**							**CSRP3**
	**CTF1**							**CTF1**
CTLA4		CTLA4			CTLA4			
CTNNA3	CTNNA3	CTNNA3		CTNNA3				
CTSB		CTSB			CTSB			
CTSL		CTSL					CTSL	
CX3CR1		CX3CR1		CX3CR1				
CXADR		CXADR			CXADR			
CYP2E1		CYP2E1			CYP2E1			
DAG1		DAG1	DAG1					
**DES**	**DES**							**DES**
DMD	DMD	DMD	DMD					
DNAJC19	DNAJC19	DNAJC19	DNAJC19					
	DOLK	DOLK	DOLK					
DSC2	DSC2	DSC2				DSC		
**DSG2**	**DSG2**							**DSG2**
	**DSP**							**DSP**
	**DTNA**							**DTNA**
EDN1		EDN1		EDN1				
EDNRA		EDNRA			EDNRA			
EDNRB		EDNRB			EDNRB			
	EMD	EMD	EMD					
ERBB2		ERBB2			ERBB2			
ESR1		ESR1		ESR1				
ESR2		ESR2			ESR2			

ESRRB		ESRRB			ESRRB			
**EYA4**	**EYA4**							**EYA4**
FAS		FAS			FAS			
FBXO32		FBXO32					FBXO32	
FHOD3		FHOD3					FHOD3	
	FHL1	FHL1	FHL1					
	FHL2	FHL2		FHL2				
FKRP	FKRP	FKRP	FKRP					
FKTN	FKTN	FKTN	FKTN					
**FLNC**	**FLNC**							**FLNC**
FN1		FN1			FN1			
FOXD4		FOXD4					FOXD4	
	FRDA/FXN	FRDA/FXN	FRDA/FXN					
	GAA	GAA	GAA					
GATA4	GATA4	GATA4				GATA4		
GATA6	GATA6	GATA6				GATA6		
**GATAD1**	**GATAD1**							**GATAD1**
GBE1		GBE1	GBE1					
GDF1		GDF1		GDF1				
GJA4		GJA4					GJA4	
	GLA	GLA	GLA					
HAND1		HAND1					HAND1	
HAVCR1		HAVCR1					HAVCR1	
	HCN4	HCN4		HCN4				
HFE	HFE	HFE		HFE				
HLA-DQB1		HLA-DQB1			HLA-DQB1			
HLA-DRB1		HLA-DRB1			HLA-DRB1			
HLA-DRB4		HLA-DRB4					HLA-DRB4	
HLA-G		HLA-G			HLA-G			
	HOPX	HOPX					HOPX	
	HRAS	HRAS	HRAS					
HSPD1		HSPD1			HSPD1			
IFIH1		IFIH1			IFIH1			
IFNG		IFNG			IFNG			
IGF1		IGF1			IGF1			
IL10		IL10			IL10			
IL17A		IL17A					IL17A	
IL17F		IL17F			IL17F			
IL1B		IL1B			IL1B			
IL2RA		IL2RA			IL2RA			
IL6		IL6			IL6			
	**ILK**							**ILK**
ITGA6		ITGA6			ITGA6			
ITGB1BP2		ITGB1BP2					ITGB1BP2	
ITGB4		ITGB4			ITGB4			
ITLN1		ITLN1					ITLN1	
ITPR2		ITPR2			ITPR2			
	**JPH2**							**JPH2**
	JUP	JUP		JUP				
KCNJ11		KCNJ11			KCNJ11			
KCNJ12		KCNJ12					KCNJ12	
KCNJ2		KCNJ2		KCNJ2				
KCNN3		KCNN3			KCNN3			
KCNQ1	KCNQ1	KCNQ1		KCNQ1				
	KRAS	KRAS	KRAS					
LAMA2	LAMA2	LAMA2	LAMA2					
**LAMA4**	**LAMA4**							**LAMA4**
LAMP2	LAMP2	LAMP2	LAMP2					
LCN2		LCN2					LCN2	
**LDB3**	**LDB3**							**LDB3**
LGALS3		LGALS3					LGALS3	
**LMNA**								**LMNA**
LRP1		LRP1			LRP1			
**LRRC10**	**LRRC10**							**LRRC10**
LRRC32		LRRC32					LRRC32	
LTBP4		LTBP4			LTBP4			
	MAP2K1	MAP2K1	MAP2K1					
	MAP2K2	MAP2K2	MAP2K2					
MAPK14		MAPK14					MAPK14	
	**MIB1**							**MIB1**
MIR199A1		MIR199A1					MIR199A1	
MIR208A		MIR208A					MIR208A	
MIR214		MIR214					MIR214	
MIR451A		MIR451A					MIR415A	
MLIP		MLIP					MLIP	

MMP1		MMP1			MMP1			
MMP10		MMP10					MMP10	
MMP14		MMP14			MMP14			
MMP3		MMP3		MMP3				
MMP7		MMP7					MMP7	
MMP9		MMP9			MMP9			
MT-TI		MT-TI	MTTI					
	MTND1	MTND1	MTND1					
	MTND5	MTND5	MTND5					
	MTND6	MTND6	MTND6					
	MTTD	MTTD	MTTD					
	MTTG	MTTG	MTTG					
	MTTH	MTTH	MTTH					
	MTTI	MTTI	MTTI					
	MTTK	MTTK	MTTK					
	MTTL1	MTTL1	MTTL1					
	MTTL2	MTTL2	MTTL2					
	MTTM	MTTM			MTTM			
	MTTQ	MTTQ			MTTQ			
	MTTS1	MTTS1			MTTS1			
	MTTS2	MTTS2			MTTS2			
CAVIN4	MURC	MURC/CAVIN					MURC/CAVIN	
MYBPC1		MYBPC1			MYBPC1			
**MYBPC3**	**MYBPC3**							**MYBPC3**
MYC		MYC			MYC			
**MYH6**	**MYH6**							**MYH6**
**MYH7**	**MYH7**							**MYH7**
**MYL2**	**MYL2**							**MYL2**
	**MYL3**							**MYL3**
	MYLK2	MYLK2		MYLK2				
MYOM1	MYOM1	MYOM1		MYOM1				
	MYOZ2	MYOZ2		MYOZ2				
**MYPN**								**MYPN**
NAMPT		NAMPT					NAMPT	
NDUFV1		NDUFV1			NDUFV1			
**NEBL**	**NEBL**							**NEBL**
**NEXN**	**NEXN**							**NEXN**
NFKB1	NFKB1	NFKB1			NFKB1			
	**NKX2-5**							**NKX2-5**
NLRP3		NLRP3			NLRP3			
NOS3		NOS3			NOS3			
**NPPA**	**NPPA**							**NPPA**
NPPB		NPPB					NPPB	
NPPC		NPPC			NPPC			
NPR2		NPR2			NPR2			
NR3C2		NR3C2			NR3C2			
	NRAS	NRAS	NRAS					
**OBSCN**								OBSCN
OSM		OSM					OSM	
PDCD1		PDCD1			PDCD1			
PDE2A		PDE2A					PDE2A	
PDE3A		PDE3A			PDE3A			
	**PDLIM3**							**PDLIM3**
PGM1		PGM1	PGM1					
	**PKP2**							**PKP2**
PLEC1	PLEC1	PLEC1	PLEC					
**PLEKHM2**	**PLEKHM2**							**PLEKHM2**
**PLN**	**PLN**							**PLN**
PNPLA2		PNPLA2	PNPLA2					
POLG		POLG	POLG					
PPP1R13L		PPP1R13L					PPP1R13L	
**PRDM16**	**PRDM16**							**PRDM16**
	PRKAG2	PRKAG2		PRKAG2				
**PSEN1**								**PSEN1**
**PSEN2**								**PSEN2**
	PTPN11	PTPN11	PTPN11					
RAF1	RAF1	RAF1	RAF1 (Noonan/Rasopathy)					
RARRES2		RARRES2					RARRES2	
**RBM20**	**RBM20**							**RBM20**
	RIT1	RIT1	RIT1 (noonan/rasopathy)					
RYR2	RYR2	RYR2		RYR2				

**SCN5A**	**SCN5A**							**SCN5A**
SDHA	SDHA		SDHA			SDHA		
SERPINE1		SERPINE1			SERPINE1			
	SGCA	SGCA	SGCA					
	SGCB	SGCB	SGCB					
**SGCD**	**SGCD**							**SGCD**
	SGCG	SGCG	SGCG					
SGK1		SGK1					SGK1	
	SLC22A5	SLC22A5	SLC22A5					
SLC25A5P8		SLC25A5P8					SLC25A5P8	
SOD2	SOD2	SOD2					SOD2	
	SOS1	SOS1	SOS1					
SPEG		SPEG	SPEG					
SRF		SRF					SRF	
STAT3		STAT3			STAT3			
SYNE1		SYNE1	SYNE1					
	SYNM	SYNM					SYNM	
TAX1BP3		TAX1BP3					TAX1BP3	
TAZ	TAZ	TAZ	TAZ					
**TBX20**	**TBX20**							**TBX20**
TBX5		TBX5			TBX5			
**TCAP**	**TCAP**							**TCAP**
TFAP2A		TFAP2A			TFAP2A			
TGFB1		TGFB1			TGFB1			
	TGFB3	TGFB3		TGFB3				
TIMP1		TIMP1					TIMP1	
TIMP4		TIMP4					TIMP4	
	TMEM43	TMEM43	TMEM43					
TMPO		TMPO					TMPO	
TNC		TNC			TNC			
TNF		TNF			TNF			
TNFRSF11B		TNFRSF11B			TNFRSF11B			
TNFRSF12A		TNFRSF12A					TNFRSF12A	
TNFSF10		TNFSF10					TITNFSF10	
TNFSF12		TNFSF12					TNFSF1	
**TNNC1**	**TNNC1**							**TNNC1**
**TNNI3**	**TNNI3**							**TNNI3**
**TNNI3K**	**TNNI3K**							**TNNI3K**
**TNNT2**	**TNNT2**							**TNNT2**
TP53		TP53			TP53			
**TPM1**	**TPM1**							**TPM1**
	TRDN	TRDN		TRDN				
TRPV2		TRPV2					TRPV2	
**TTN**	TTN							**TTN**
	TTR	TTR		TTR				
TWIST1		TWIST1			TWIST			
TXNRD2	TXNRD2	TXNRD2	TXNRD2					
**VCL**	**VCL**							**VCL**
VEGFA		VEGFA			VEGFA			
ZASP		ZASP					REPEAT - SEE LDB3	
ZBTB17	ZBTB17	ZBTB17					ZBTB17	
**TOTAL: 204**	**TOTAL: 63** (125-62)	**TOTAL: 216**	**TOTAL: -55**	**TOTAL: -26**	**TOTAL: -70**	**TOTAL: -5**	**TOTAL: -60**	**TOTAL: 51**
**TOTAL BEFORE REMOVED: 267**		**TOTAL AFTER REMOVED: 51**	**TOTAL OF ALL REMOVED CATEGORIES: 216**					**TOTAL FINAL LIST: 51**

Supplemental Table II

DCM Classification	Curated Genes	USA: Invitae	USA: EGL Genetic Diagnostics	USA: Prevention Genetics	USA: Cincinnati's Childrens Hospital	USA: Ambry Genetics	Finland: Blueprint Genetics	USA: Fulgent Genetics	USA: Washington University	Spain: Health in Code	USA: Knight Diagnostic Laboratories	Spain: CGC Genetics	USA: Gene Dx	Germany: CeGaT GmbH	Germany: MGZ - Medical Genetics Center	Canada: LifeLabs Genetics	USA: Sema4	Panel Totals	% Panels Present
Definitive	BAG3	1	1	1	1	1	1	1	1	1	1	1	1	1	1	1	1	16	100.0%
	DES	1	1	1	1	1	1	1	1	1	1	1	1	1	1	1	1	16	100.0%
	LMNA	1	1	1	1	1	1	1	1	1	1	1	1	1	1	1	1	16	100.0%
	MYH7	1	1	1	1	1	1	1	1	1	1	1	1	1	1	1	1	16	100.0%
	PLN	1	1	1	1	1	1	1	1	1	1	1	1	1	1	1	1	16	100.0%
	RBM20	1	1	1	1	1	1	1	1	1	1	1	1	1	1	1	1	16	100.0%
	SCN5A	1	1	1	1	1	1	1	1	1	1	1	1	1	1	1	1	16	100.0%
	TTN	1	1	1	1	1	1	1	1	1	1	1	1	1	1	1	1	16	100.0%
	TNNC1	1	1	1	1	1	1	1	1	1	0	1	1	1	1	1	1	15	93.8%
	TNNT2	1	1	1	1	1	1	1	1	1	0	1	1	1	1	1	1	15	93.8%
	FLNC	1	0	1	1	1	1	1	1	1	0	1	1	1	0	0	1	12	75.0%
Strong	DSP	1	1	1	1	0	1	1	0	1	1	1	1	1	1	1	1	14	87.5%
Moderate	ACTC1	1	1	1	1	1	1	1	1	1	1	1	1	1	1	1	1	16	100.0%
	TNNI3	1	1	1	1	1	1	1	1	1	1	1	1	1	1	1	1	16	100.0%
	TPM1	1	1	1	1	1	1	1	1	1	1	1	1	1	1	1	1	16	100.0%
	VCL	1	1	1	1	1	1	1	1	1	1	1	1	1	1	1	1	16	100.0%
	ACTN2	0	1	1	1	1	1	1	1	1	1	1	1	1	1	1	1	15	93.8%
	NEXN	1	1	1	1	1	0	1	1	1	1	1	1	1	1	1	1	15	93.8%
	JPH2	0	0	0	1	0	1	1	0	0	0	0	0	1	0	0	0	4	25.0%
Limited	ABCC9	1	1	1	1	1	1	1	1	1	1	1	1	1	1	1	1	16	100.0%
	LDB3	1	1	1	1	1	1	1	1	1	1	1	1	1	1	1	1	16	100.0%
	MYBPC3	1	1	1	1	1	1	1	1	1	1	1	1	1	1	1	1	16	100.0%
	MYH6	1	1	1	1	1	1	1	1	1	1	1	1	1	1	1	1	16	100.0%
	TCAP	1	1	1	1	1	1	1	1	1	1	1	1	1	1	1	1	16	100.0%
	ANKRD1	1	1	1	1	1	0	1	1	1	1	1	1	1	1	1	1	15	93.8%
	CSRP3	1	1	1	1	1	0	1	1	1	1	1	1	1	1	1	1	15	93.8%
	LAMA4	1	1	1	1	1	1	1	0	1	1	1	1	1	1	1	1	15	93.8%
	MYPN	1	1	1	1	1	0	1	1	1	1	1	1	1	1	1	1	15	93.8%
	DSG2	1	1	1	1	0	1	1	0	1	1	1	1	1	1	1	1	14	87.5%
	GATAD1	1	1	1	1	0	0	1	1	1	1	1	1	1	1	1	1	14	87.5%
	ILK	1	0	1	1	0	1	1	1	1	1	1	1	1	1	1	1	14	87.5%
	NEBL	1	1	1	1	0	0	1	1	1	1	1	1	1	0	1	1	13	81.3%
	PRDM16	1	1	1	1	0	1	1	0	1	0	1	1	1	1	1	1	13	81.3%
	SGCD	0	1	1	1	0	0	1	1	1	1	1	1	1	1	1	1	13	81.3%
	EYA4	1	0	1	1	0	0	1	1	1	1	1	0	0	1	1	1	11	68.8%
	NKX2-5	1	0	1	1	1	1	1	1	1	0	1	1	0	0	0	0	10	62.5%
	TBX20	0	0	0	1	1	1	1	1	1	0	0	1	0	1	1	0	9	56.3%
	DTNA	0	0	0	1	0	0	1	0	1	0	0	1	1	0	0	0	5	31.3%
	MYL2	0	0	0	1	0	0	1	0	1	0	1	0	1	0	0	0	5	31.3%
	CTF1	1	0	0	0	0	0	1	1	0	0	1	0	0	0	0	0	4	25.0%
	PLEKHM2	1	0	0	0	0	1	0	1	0	0	0	0	0	0	0	0	3	18.8%
	PSEN2	0	0	0	0	0	0	0	0	1	0	1	0	0	1	0	0	3	18.8%
	TNNI3K	0	0	0	0	1	1	0	0	1	0	0	0	0	0	0	0	3	18.8%
	OBSCN	0	0	0	0	0	0	0	0	1	0	0	0	0	0	0	1	2	12.5%
Disputed	PDLIM3	1	1	1	1	0	0	1	1	1	1	1	0	1	1	1	0	12	75.0%
	PKP2	1	0	1	1	0	1	1	0	1	1	1	0	1	1	1	1	12	75.0%
